# Sodium Glucose Co-Transporter-2 inhibitors inpatients with systemic sclerosis with or without heart failure

**DOI:** 10.1016/j.amjmed.2025.08.009

**Published:** 2025-08-12

**Authors:** Silvio Nunes Augusto, David C. Kaelber, Soumya Chatterjee, W.H. Wilson Tang

**Affiliations:** a Cardiovascular and Metabolic Sciences, Cleveland Clinic Research, Cleveland Clinic, Cleveland, OH, USA; b The Center for Clinical Informatics Research and Education, The MetroHealth System, Cleveland, OH, USA; c The Departments of Internal Medicine, Pediatrics, and Population and Quantitative Health Sciences, Case Western Reserve University, Cleveland, OH, USA; d Department of Rheumatic and Immunologic Disease, Medical Specialty Institute, Cleveland Clinic, Cleveland, OH, USA; e Cleveland Clinic Lerner College of Medicine of Case Western Reserve University, Cleveland, OH, USA; f Department of Cardiovascular Medicine, Heart, Vascular and Thoracic Institute, Cleveland Clinic, Cleveland, OH, USA

**Keywords:** SGLT2 inhibitors, Systemic sclerosis, Mortality, Hospitalization, Stroke

## Abstract

**BACKGROUND:**

Patients with systemic sclerosis are at heightened risk of developing heart failure. Sodium-glucose cotransporter 2 inhibitors (SGLT2i) have shown cardio-renal benefits in diverse populations with cardio-renal dysfunction, but their impact on outcomes in patients with systemic sclerosis has not been described.

**METHODS:**

This retrospective cohort study used the Research Network of the TriNetX platform to compare outcomes between patients with systemic sclerosis prescribed with SGLT2 inhibitors versus those without SGLT2i prescription, using data from January 1, 2013, to May 6, 2025. Following propensity score matching, each cohort included 1,402 patients with balanced baseline characteristics (standardized mean differences <0.2). The primary outcome was all-cause mortality. Secondary outcomes included first-time heart failure diagnosis, acute heart failure, acute myocardial infarction, hospitalization, stroke, cardiac arrest, and chronic kidney disease.

**RESULTS:**

SGLT2i prescription was associated with a significantly lower risk of all-cause mortality (Hazard Ratio [HR] 0.54, 95 % confidence interval [CI] 0.44-0.66), stroke (HR 0.64, 95 % CI 0.42-0.96), and hospitalization (HR 0.76, 95 % CI 0.66-0.86). Notably, patients without a history of heart failure (HR 0.62, 95 % CI 0.45-0.87) and patients with a history of heart failure also had a lower risk of hospitalization (HR 0.76, 95 % CI 0.57-1.00). No significant differences were observed in the risks of major adverse cardiovascular events, acute myocardial infarction, or chronic kidney disease.

**CONCLUSION:**

In this real-world analysis, SGLT2 inhibitors were associated with reduced mortality, stroke, and hospitalization in patients with systemic sclerosis, supporting their potential therapeutic role in this population.

## Introduction

Sodium-glucose cotransporter-2 inhibitors (SGLT2i) have demonstrated significant reductions in hospitalization rates for heart failure and cardiovascular events across diverse populations, with evidence further suggesting that SGLT2i lower all-cause mortality and hospitalization compared to other glucose-lowering drugs.^1–7^ Furthermore, continued use of SGLT2i during hospitalization has been associated with reduced in-hospital mortality and shorter lengths of stay without increasing the risk of acute kidney injury.^8^ Studies have shown that patients with systemic sclerosis have a 2-3 fold higher risk of developing heart failure than those without the condition even after adjusting for other risk factors, and is a leading cause or mortality in this population.^9–15^ Cardiac involvement in systemic sclerosis has been attributed to progressive myocardial fibrosis and inflammation, leading to left ventricular diastolic and occasionally systolic dysfunction. This risk is particularly elevated in patients with diffuse cutaneous disease, in older men, with tendon friction rubs, or with specific sero-logical markers.^15–17^ The risk of developing heart failure in systemic sclerosis increases over time, with some cohorts showing 10-year cumulative incidence rates nearing 10 %.^10^

Given the strong evidence from recent guidelines and clinical trials showing that SGLT2i offer cardiovascular and renal protection in at-risk and heart failure populations, independent of diabetes, patients with systemic sclerosis may also potentially benefit from this therapy. However, specific data on the impact of SGLT2i in patients with systemic sclerosis is lacking. While the cardio-renal protective benefits of SGLT2i are well-documented in broader populations, including those with significant comorbidities, no studies have directly evaluated their effects on hospitalization rates in patients with systemic sclerosis. In this study, we sought to investigate whether patients with systemic sclerosis with or without heart failure taking SGLT2i are less likely to suffer adverse clinical outcomes.

## Methods

### Data Availability.

The data, analytic methods, and study materials were accessed through the Research Network of the TriNetX platform, which includes aggregated, de-identified electronic health records (EHRs) from 106 healthcare organizations (HCOs). This study utilized data from January 1, 2013, to May 6, 2025 that coincides with the clinical availability of SGLT2i. Institutional Review Board approval was not required as the study involved a secondary analysis of de-identified data, exempt from informed consent per the HIPAA Privacy Rule Section §164.514(a). The de-identification process was certified by a qualified expert according to Section §164.514(b)(1), with the most recent certification completed in December 2020. This observational retrospective cohort study followed the STROBE guidelines.^18^

### Study population.

**Fig. 1:**
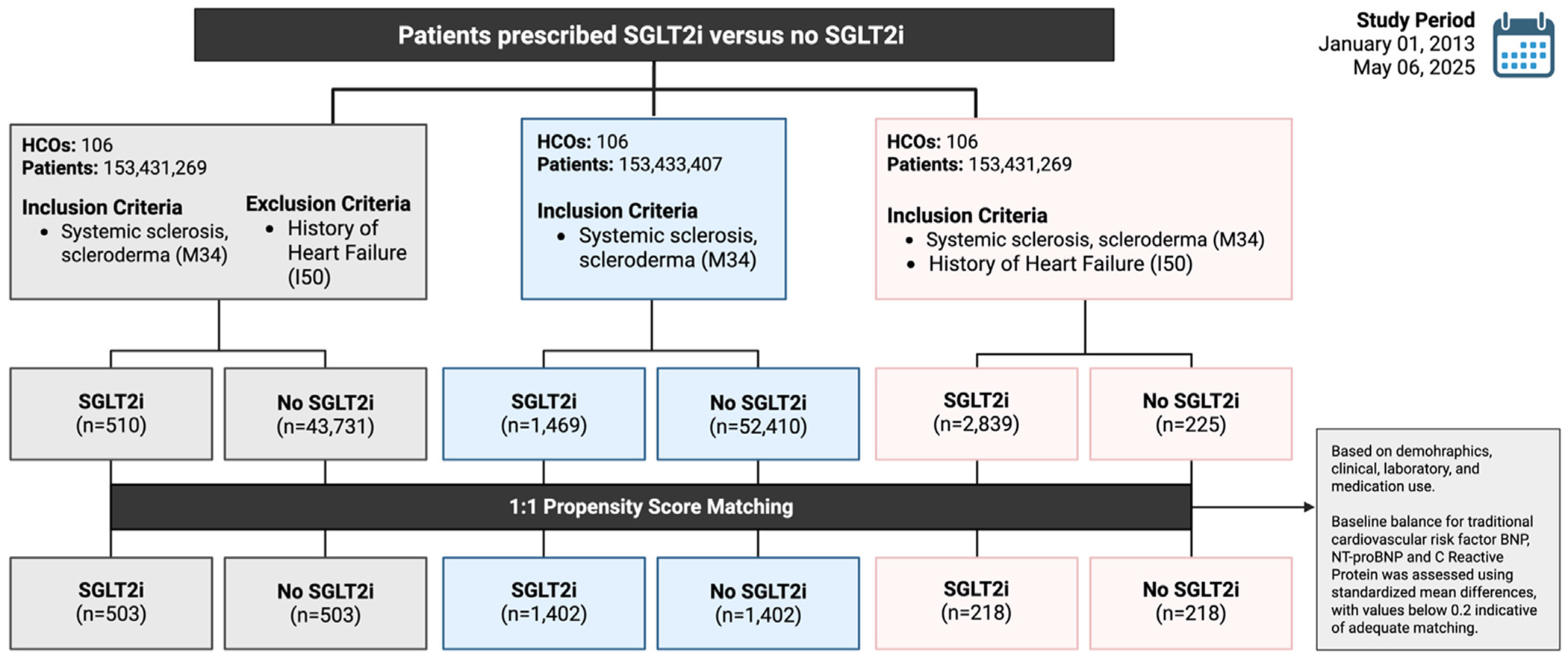
STROBE Diagram: This study utilized data from January 1, 2013, to May 6, 2025. The STROBE diagram shows the inclusion criteria for the primary cohort and the two sub-cohorts and describes propensity score matching variables up to outcome analysis. Before propensity score matching, the SGLT2i cohort consisted of 1,469 patients, while the cohort without SGLT2i included 52,410 patients. Following matching, each cohort included 1,402 patients, achieving balance across all baseline variables with standardized mean differences all <0.2.

Fig. 1 shows the flow chart of patient selection and describes propensity score matching variables up to outcome analysis. The study included patients older than 18 years, with a follow-up period of 5 years post-index date. In the SGLT2i cohort, the inclusion criteria included patients with at least one encounter diagnosis of systemic sclerosis (ICD-10-CM: M34) and the first SGLT2i prescription occurred at least 1 day after any instance of systemic sclerosis during the study window (ICD-10-CM: A10BK, sodium-glucose co-transporter; RxNorm: 1992672, ertugliflozin; 1488564, dapagliflozin; 1373458, canagliflozin; 1545653, empagliflozin; 2638675, sotagliflozin; 2627044, bexagliflozin). The cohort without SGLT2i also included patients with systemic sclerosis but who were never prescribed any SGLT2i medications.

Two sub-cohort studies were analyzed to further explore the benefits of SGLT2i in a subgroup of systemic sclerosis patients with and without any ICD-10 associated heart failure encounter diagnoses. Baseline characteristics of the primary cohort were collected no longer than 1 year before the index events. Propensity score matching was performed based on the following variables: In the demographics category, patients were matched on age, sex, race, and ethnicity. In the diagnosis category, patients were matched on history of smoking (%), hypertension (%), hyperlipidemia (%), and type 2 diabetes mellitus (%). In the medication category, patients were matched on beta-blocking agents (%), loop diuretics (%), diuretics (%), angiotensin converting enzyme inhibitors (%), antiarrhythmics (%), antilipemic agents (%), analgesics (%), angiotensin II receptor blockers (%), aspirin (%), and corticosteroids (%). In the laboratory category the most recent lab values were included, and patients were matched on BMI (kg/m^2^), body weight (kg), left ventricular ejection fraction (LVEF) (%), triglycerides (mg/dL), cholesterol (mg/dL), HDL cholesterol (mg/dL), LDL cholesterol (mg/dL), glucose (mg/dL), hemoglobin (g/dL), bicarbonate (mmol/L), iron (mg/dL), sodium (mmol/L), potassium (mmol/L), chloride (mmol/L), urea nitrogen (mg/dL), creatinine (mg/dL), estimated glomerular filtration rate (eGFR) (mL/min/1.73m^2^), B-type natriuretic peptide (pg/mL), aminoterminal pro B-type natriuretic peptide (pg/mL), and C-reactive protein (mg/L).

### Study endpoints & statistical analysis.

Clinical outcomes were identified using ICD-10 codes and included all-cause mortality, defined by recorded death in demographic data; major adverse cardiovascular events, which composed of acute myocardial infarction (I21), nontraumatic intrace-rebral hemorrhage (I61), cerebral infarction (I63), and sequelae of cerebrovascular disease (I69); stroke, including cerebral infarction (I63), acute cerebrovascular insufficiency (I67.81), and cerebral atherosclerosis (I67.2); acute myocardial infarction (I21); chronic kidney disease (N18); cardiac arrest (I46); a hospitalization composite when a Common Procedural Terminology (CPT) code for an encounter for the evaluation and management of a patient exceeded 40 minutes (99221), 55 minutes (99222), or 75 minutes (99223) was used. For all outcomes, patients with the respective diagnosis before the time window were excluded from survival and risk analyses, except for hospitalization. Risk differences, risk ratios, and odds ratios were calculated for each outcome with accompanying 95 % confidence intervals. Kaplan-Meier survival analyses were conducted to estimate survival probabilities and hazard ratios (HR) were derived using Cox proportional hazards models. Baseline balance was assessed using standardized mean differences, with values below 0.2 indicative of adequate matching.

## Results

### Baseline characteristics.

Before propensity score matching, the SGLT2i cohort consisted of 1,469 patients, while the cohort without SGLT2i included 52,410 patients. Following matching, each cohort included 1,402 patients, achieving balance across all baseline variables with standardized mean differences all <0.2 (Table 1).

### Clinical outcomes.

**Fig. 2:**
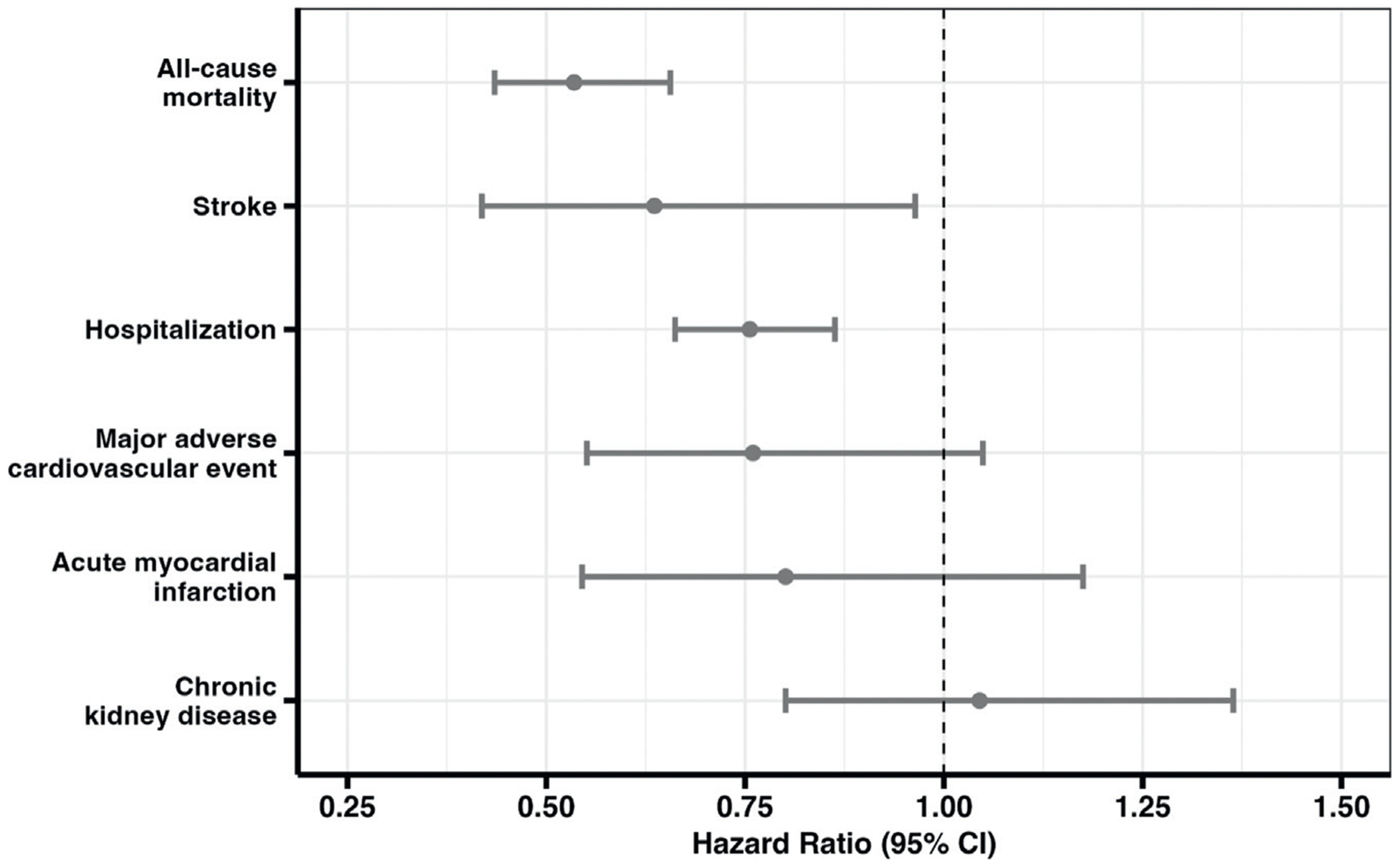
Forest Plot Hazard-Ratio for primary cohort analysis. Figure 2 shows that at the end of the five-year follow-up, patients in the SGLT2i cohort experienced a significantly lower risk of all-cause mortality (Hazard Ratio [HR] 0.54, 95 % confidence interval [CI] 0.44-0.66), stroke (HR 0.64, 95 %CI 0.42-0.96), and hospitalization (HR 0.76, 95 %CI 0.66-0.86) compared with patients in the without SGLT2i cohort. No statistically significant differences were observed for major adverse cardiovascular events (HR 0.76, 95 %CI 0.55-1.05), acute myocardial infarction (HR 0.80, 95 %CI 0.5-51.18), and chronic kidney disease (HR 1.05, 95 %CI 0.80-1.36).

**Fig. 3:**
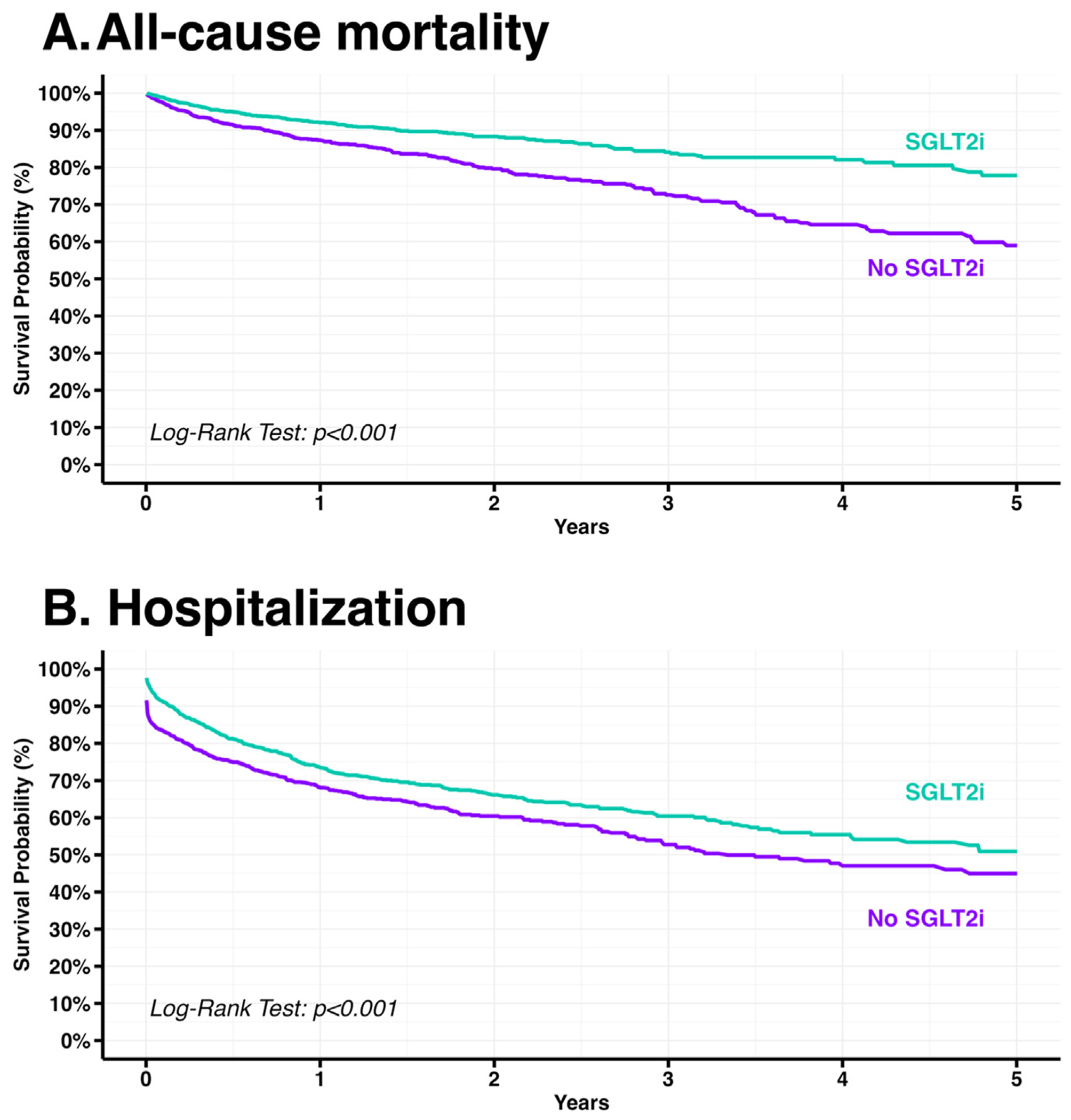
Kaplan-Meier curves for all-cause mortality and hospitalization. (**A**) shows the Kaplan-Meier curve for all-cause mortality (log-rank test *P*<0.001), whereas (**B**) shows the Kaplan-Meier curve for hospitalization (log-rank test *P*<0.001).

**Fig. 4:**
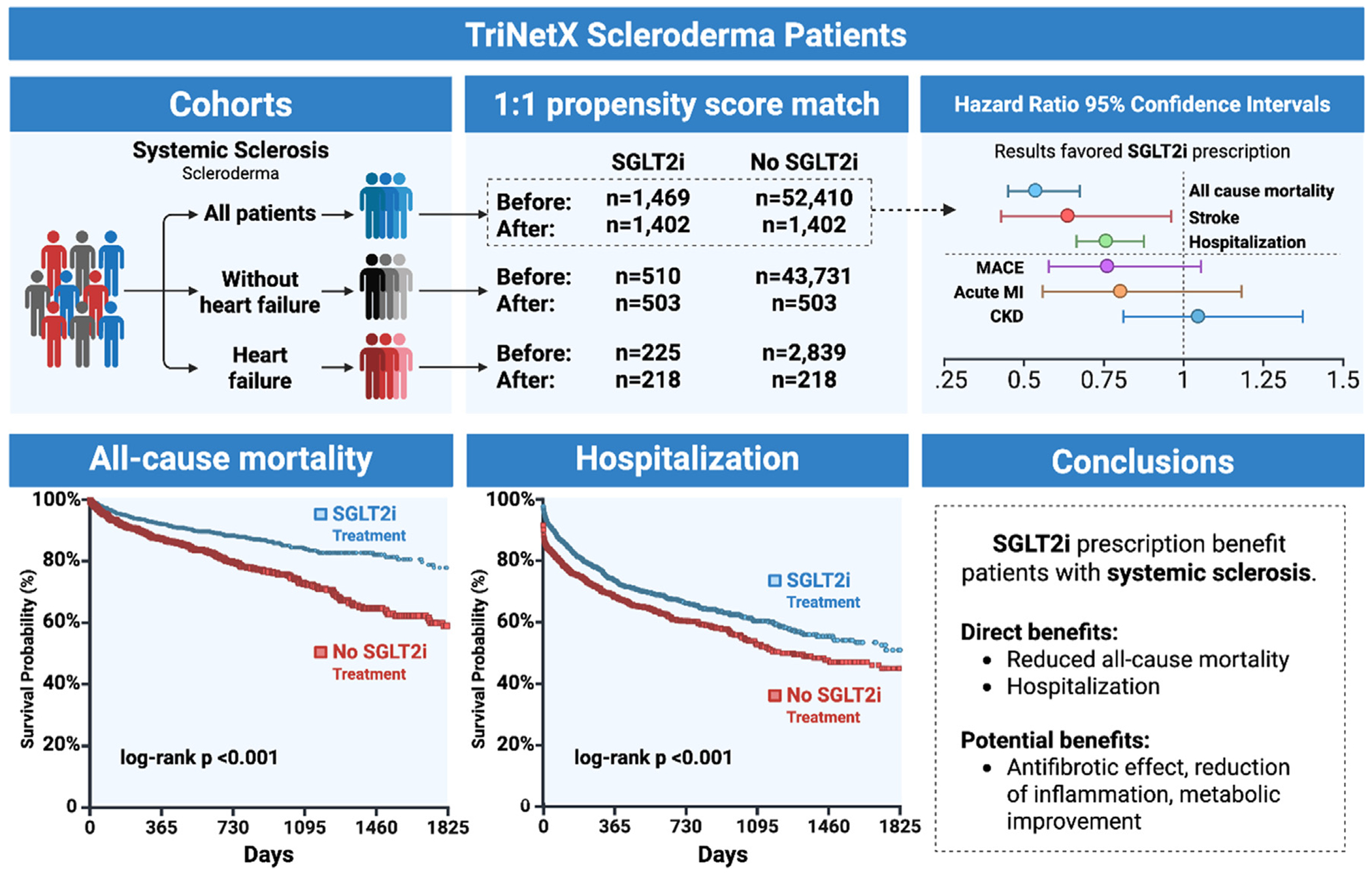
Central Illustration. One primary cohort and two sub-cohort of patients were investigated. Patients were 1:1 propensity score-matched, and hazard ratio 95 % confidence intervals are shown for all-cause mortality, stroke, hospitalization, major adverse cardiovascular event, acute myocardial infarction, and chronic kidney disease. Kaplan-Meier survival curves show reduced all-cause mortality and reduced hospitalization in patients prescribed SGLT2i medication.

Table 2 and Fig. 2 show hazard ratios for the primary cohort analysis. At the end of the five-year follow-up, patients in the SGLT2i cohort experienced a significantly lower risk of all-cause mortality (HR 0.54, 95 % confidence interval [CI] 0.44-0.66), stroke (HR 0.64, 95 %CI 0.42-0.96), and hospitalization (HR 0.76, 95 %CI 0.66-0.86) compared with patients in the cohort without SGLT2i prescription. No statistically significant differences were observed for major adverse cardiovascular events (HR 0.76, 95 %CI 0.55-1.05), acute myocardial infarction (HR 0.80, 95 %CI 0.55-1.18), chronic kidney disease (HR 1.05, 95 %CI 0.80-1.36). Fig. 3 further shows the two Kaplan-Meier curves for all-cause mortality and hospitalization, which are also part of the central illustration in Fig. 4.

When stratified by a history of heart failure diagnoses, outcome patterns varied between subgroups. Among patients without a history of heart failure, SGLT2i prescriptions were associated with a significantly lower risk of hospitalization (HR 0.62, 95 % CI 0.45-0.87). Although the hazard ratios for all-cause mortality (HR 0.64, 95 %CI 0.36-1.12) and stroke (HR 0.67, 95 %CI 0.31-1.44) also favored SGLT2i prescription, they did not reach statistical significance. Similarly, no significant differences were observed for major adverse cardiovascular events (HR 0.83, 95 %CI 0.44-1.56), acute myocardial infarction (HR 1.09, 95 %CI 0.40-3.00), or chronic kidney disease (HR 0.97, 95 %CI 0.56-1.68) ICD-related encounter diagnoses.

Among patients with prior heart failure diagnoses, SGLT2i prescriptions were also associated with a lower risk of hospitalization (HR 0.76, 95 %CI 0.57-1.00). While the point estimates for all-cause mortality (HR 0.66, 95 %CI 0.42-1.03) and stroke (HR 0.38, 95 %CI 0.10-1.46) favored SGLT2i prescription, they did not reach statistical significance. No significant differences were observed for acute myocardial infarction (HR 0.98, 95 %CI 0.39-2.46), major adverse cardiovascular events (HR 1.43, 95 %CI 0.60-3.38), or chronic kidney disease (HR 1.37, 95 %CI 0.74-2.52) ICD-related encounter diagnoses.

## Discussion

Our analysis provides a hypothesis-generating observation that patients prescribed SGLT2i medications may experience overall benefit against all-cause mortality. In addition, our subgroup analysis also revealed that patients (with and without heart failure) may benefit from fewer hospitalizations. These results are consistent with recent clinical trials and observational studies showing that SGLT2i significantly improve clinical outcomes and reduce hospitalizations across different patient populations.^19,20^ For instance, the EMPA-REG OUTCOME trial showed a 34 % reduction in the combined endpoint of cardiovascular death or hospitalization for heart failure with empagliflozin.^21^ Similarly, the CANVAS program reported a 33 % reduction in heart failure hospitalization with canagliflozin.^22^ A nationwide cohort study also found that continued use of SGLT2i during hospitalization was associated with a 45 % lower in-hospital mortality rate and a decreased length of stay without an increased risk of acute kidney injury.^8^ Furthermore, a systematic review and network meta-analysis confirmed that SGLT2i, including canagliflozin, dapagliflozin, and empagliflozin, are associated with reductions in all-cause mortality, cardiovascular mortality, and hospitalization for heart failure.^23^ On the other hand, kidney involvement is common in patients with diffuse systemic sclerosis (especially with renal crisis, which can be exacerbated by drugs such as angiotensin converting enzyme inhibitors). It is, therefore, reassuring to see that SGLT2i was not associated with an increased risk of chronic kidney disease, although we did not observe a reduction in incident chronic kidney disease like the reno-protective effects observed in patients with established chronic kidney disease.^24^ Further studies are warranted to investigate whether SGLT2i provides long-term reno-protection.

Even though the precise mechanisms by which SGLT2i confer these benefits remain to be determined, it is recognized that SGLT2i can attenuate inflammation and potentially modulate T-cell activity.^25^ These benefits extend beyond glucose-lowering, thereby offering potential advantages in attenuating disease progression in autoimmune diseases. Furthermore, reduced cardiac stress and improved renal outcomes may further contribute to reduced hospitalization and rehospitalization rates in patients with HFrEF, even though we do not have any specific evidence in our analysis to support these effects.^26^

### Limitations.

This study utilized the TriNetX platform, which carries inherent limitations associated with retrospective analyses of EHR data. Notably, residual or unmeasured confounding cannot be entirely ruled out due to the observational nature of the data. As the study relies on pre-existing EHR data that was not originally intended for research, the accuracy and completeness of the information cannot be guaranteed. With regards to the inclusion criteria, the diagnosis of systemic sclerosis was based on clinician documentation and we cannot confirm the American College of Rheumatology Diagnostic Criteria as not all parameters are easily accessible in the TriNetX platform. Because we do not have access to patient follow-up or to individual patient data, it is not possible to determine the course of systemic sclerosis or its development accurately. The outcome of all-cause mortality should also be interpreted with caution, as deaths occurring outside the hospital may not be promptly captured in the system until verified via Social Security records. Although hospitalization reached statistical significance, it was defined as a composite outcome using three ICD codes not specific to heart failure, limiting its clinical interpretability. Still, this does not undermine our central finding: patients with systemic sclerosis, regardless of heart failure status, may benefit from SGLT2i therapy. While we performed propensity matching for a broad range of medications, we lacked detailed access to patient histories to fully assess potential confounding from drug interactions. Additionally, we are unable to evaluate medication adherence or dose-response effects, as the observational nature of our study relies on prescription codes, not compliance; however, this may lead to an underestimation of the true benefit of SGLT2i, as real-world non-adherence could dilute the observed treatment effect.

## Conclusion

In conclusion, our propensity-matched analysis shows that SGLT2i prescriptions were associated with significantly lower risks of all-cause mortality and hospitalization in patients with systemic sclerosis over a five-year follow-up period.

## Figures and Tables

**Table 1:** Baseline characteristics between cohorts before and after propensity matching.

	Before Propensity Score Matching				After Propensity Score Matching			
	SGLT2i (n=1,469)	No SGLT2i (52,410)	*P* Value	Std diff.	SGLT2i (n=1,402)	No SGLT2i (n=1,402)	*P* Value	Std diff.
Demographics								
Age (years)	66.1 ± 12.6	62.8 ± 16.1	<0.001	0.226	66.0 ± 12.6	66.9 ± 14.8	0.064	0.070
Male	25 %	16%	<0.001	0.213	24 %	25 %	0.662	0.017
White	62 %	66 %	0.001	0.086	63 %	63 %	0.907	0.004
Not Hispanic or Latino	75 %	67 %	<0.001	0.17	75 %	76 %	0.542	0.023
Clinical								
History of Smoking	0.166	0.042	<0.001	0.416	0.165	0.162	0.838	0.008
BMI (kg/m^2^)	30.3 ± 8.4	27.5 ± 7.1	<0.001	0.36	30.1 ± 8.3	29.0 ± 7.8	0.003	0.131
Body weight (kg)	179.3 ± 54.7	163.2 ± 46.6	<0.001	0.316	178.2 ± 53.9	173.9 ± 54.7	0.071	0.079
Hyperlipidemia (%)	41%	9%	<0.001	0.799	39%	38%	0.613	0.019
Hypertension (%)	60 %	17 %	<0.001	0.981	58 %	61 %	0.106	0.061
Type 2 diabetes (%)	44 %	6%	<0.001	1	42 %	44 %	0.341	0.036
BP Systolic (mmHg)	72 %	39 %	0.436	0.023	71 %	73 %	0.008	0.118
BP Diastolic (mmHg)	72 %	39 %	<0.001	0.126	71 %	73 %	0.323	0.044
Left Ventricular Ejection Fraction (LVEF) (%)	54.9 ± 13.9	59.6 ± 10.0	<0.001	0.389	56.0 ± 13.6	54.2 ± 13.6	0.371	0.128
Laboratory								
Total cholesterol (mg/dL)	162.8 ± 49.5	178.6 ± 46.0	<0.001	0.331	164.6 ± 49.6	159.2 ± 49.5	0.071	0.108
HDL Cholesterol (mg/dL)	47.8 ± 17.4	53.5 ± 19.0	<0.001	0.313	48.1 ± 17.7	49.4 ± 18.6	0.219	0.073
LDL Cholesterol (mg/dL)	87.2 ± 37.9	100.5 ± 36.6	<0.001	0.357	88.4 ± 37.7	86.3 ± 36.2	0.356	0.055
Sodium (mg/dL)	138.7 ± 3.3	138.9 ± 3.2	0.039	0.062	138.6 ± 3.3	138.6 ± 3.2	0.976	0.001
Potassium (mg/dL)	4.2 ± 0.5	4.1 ± 0.5	0.001	0.101	4.2 ± 0.5	4.2 ± 0.5	0.807	0.011
Chloride (mg/dL)	102.8 ± 4.6	103.3 ± 4.6	0.001	0.107	102.9 ± 4.6	103.0 ± 4.3	0.512	0.029
Glucose (mg/dL)	126.7 ± 60.9	102.7 ± 33.8	<0.001	0.488	125.9 ± 60.4	118.6 ± 47.4	0.002	0.135
BNP (pg/mL)1	712.0 ± 1554.7	412.4 ± 1020.1	<0.001	0.228	672.5 ± 1440.8	544.6 ± 822.4	0.19	0.109
NT-proBNP (pg/mL)	3490.8 ± 7645.9	2411.5 ± 6492.6	0.011	0.152	3300.4 ± 7285.7	3329.9 ± 8027.7	0.965	0.004
C Reactive Protein (mg/L)	21.1 ± 36.0	14.2 ± 31.2	<0.001	0.204	21.0 ± 36.6	21.6 ± 40.0	0.844	0.014
Bicarbonate (mmol/L)	25.5 ± 3.8	25.7 ± 3.4	0.097	0.049	25.5 ± 3.8	25.6 ± 3.7	0.523	0.028
Iron (*μ*g/dL)	55.5 ± 34.9	63.8 ± 42.3	<0.001	0.214	56.9 ± 35.9	54.9 ± 33.8	0.475	0.056
Urea Nitrogen (mg/dL)	21.9 ± 12.8	17.0 ± 10.6	<0.001	0.425	21.7 ± 12.7	21.8 ± 13.7	0.909	0.005
Creatinine (mg/dL)	1.1 ± 0.5	1.0 ± 1.9	0.110	0.066	1.1 ± 0.5	1.3 ± 1.4	<0.001	0.177
GFR (CKD-EPI) (mL/min/1.73m^2^)	69.2 ± 27.3	84.2 ± 63.7	<0.001	0.306	69.7 ± 27.2	76.5 ± 240.4	0.385	0.039
Medications								
Beta Blockers (%)	45 %	11 %	<0.001	0.829	43 %	42 %	0.703	0.014
Loop Diuretics (%)	47 %	8%	<0.001	0.993	45 %	43 %	0.138	0.056
Diuretics (%)	58 %	13 %	<0.001	1.091	57 %	56 %	0.594	0.020
ACE Inhibitors (%)	20%	6%	<0.001	0.423	19 %	20 %	0.602	0.020
Antiarrhythmics (%)	46 %	13 %	<0.001	0.771	44 %	46 %	0.544	0.023
Lipid-lowering agents (%)	50 %	12 %	<0.001	0.919	49 %	47 %	0.473	0.027
Analgesics (%)	65 %	29 %	<0.001	0.767	63 %	65 %	0.408	0.031
Angiotensin II Inhibitors (%)	31 %	5%	<0.001	0.714	29 %	29 %	0.771	0.011
Aspirin (%)	31 %	8%	<0.001	0.624	30 %	29 %	0.741	0.012
Corticosteroids (%)	55 %	23 %	<0.001	0.683	54 %	56 %	0.306	0.039

**Table 2:** Effects of SGLT2i on clinical outcomes in propensity-matched patients with systemic sclerosis.

Outcomes		Kaplan-Meier log-rank test					
	Hazard Ratio (95 %CI)	X^2^	df	*P* Value	Risk Difference (95 %CI)	Risk Ratio (95 %CI)	Odds Ratio (95 %CI)
All-cause mortality	0.54 (0.43, 0.66)	37.061	1	<0.001	−0.07 (−0.10, −0.05)	0.59 (0.49, 0.72)	0.54 (0.44, 0.68)
Hospitalization	0.76 (0.66, 0.86)	17.076	1	<0.001	−0.04 (−0.08, −0.01)	0.88 (0.79, 0.98)	0.82 (0.70, 0.97)
Stroke	0.64 (0.42, 0.96)	4.619	1	0.032	−0.01 (−0.03, 0.00)	0.71 (0.47, 1.08)	0.71 (0.46, 1.08)
Major adverse cardiac events	0.76 (0.55, 1.05)	2.798	1	0.094	−0.01 (−0.03, 0.01)	0.85 (0.63, 1.17)	0.84 (0.60, 1.18)
Acute myocardial infarction	0.80 (0.55, 1.18)	1.294	1	0.255	−0.01 (−0.02, 0.01)	0.89 (0.61, 1.29)	0.88 (0.59, 1.30)
Chronic kidney disease	1.04 (0.80, 1.36)	0.106	1	0.745	0.02 (−0.01, 0.05)	1.21 (0.94, 1.55)	1.24 (0.93, 1.64)

## CLINICAL SIGNIFICANCE

- Patients with systemic sclerosis and prescription of sodium-glucose co-transporter 2 inhibitors (SGLT2i) were associated with lower risk for all-cause mortality, hospitalization, and stroke in the whole cohort.
- The subset analysis of patients with and without heart failure also showed that the prescription of SGLT2i was associated with reduced risk in hospitalizations.
- Our results support evidence that SGLT2i medication can be beneficial for patients with and without heart failure.
